# Interpretable machine learning classification of cedar and cypress pollen on routine Durham slides for environmental exposure assessment

**DOI:** 10.3389/falgy.2026.1805985

**Published:** 2026-05-07

**Authors:** Nobuyoshi Suzuki, Kenjiro Sugiyama, Katsuhiko Kobayashi, Hisakuni Fukuoka, Yutaka Takumi

**Affiliations:** 1 Nanohana ENT Clinic, Matsumoto, Nagano, Japan; 2 Department of Otorhinolaryngology-Head and Neck Surgery, Shinshu University School of Medicine, Matsumoto, Nagano, Japan; 3 Kobayashi ENT Clinic, Matsumoto, Nagano, Japan; 4 Fukuoka ENT Vertigo Clinic, Nagano, Nagano, Japan

**Keywords:** aerobiology, burst pollen, durham method, image analysis, interpretable machine learning, pollen monitoring, support vector machine

## Abstract

Accurate discrimination of *Cryptomeria japonica* (cedar) and *Chamaecyparis obtusa* (cypress) pollen on routine Durham slides is clinically and environmentally important, because local pollen counts influence patient visits and regional exposure assessment. However, manual counting is time-consuming and often complicated by debris, air bubbles, and burst pollen, which is morphologically distinct from intact grains. In this methodological proof-of-concept study, we evaluated whether machine learning could classify particles cropped from real-world Durham slides collected under routine field conditions. We collected five routine slides from two sites in Nagano Prefecture and obtained 1,480 particle images categorized into five classes: cedar, cypress, burst cedar, burst cypress, and dust/miscellaneous artifacts. We extracted interpretable morphological and textural descriptors and trained a support vector machine using nested 5-fold stratified cross-validation. Using an optimized, interpretable feature set, the model achieved a macro-F1 score of 0.833 ± 0.025 and an overall accuracy of 0.863. Classification of intact cedar and cypress pollen was good, whereas discrimination between the two burst classes was more difficult. Misclassifications were concentrated mainly between cedar and cypress, and between burst cedar and morphologically similar particles such as burst cypress or dust. In contrast, dust was rarely misclassified as intact cedar or intact cypress. One-vs.-rest ROC-AUC values were high across classes (0.93–0.98), although performance was lower for burst pollen than for intact pollen. Among the extracted descriptors, size-related features, particularly area and radius, contributed most strongly to classification. These findings show that interpretable machine learning can distinguish intact cedar and cypress pollen on routine Durham slides under real-world conditions, while burst pollen remains a major source of classification difficulty. Further refinement of feature design for burst pollen will be necessary for routine application.

## 1 Introduction

Seasonal allergic rhinitis caused by airborne pollen—particularly that from *Cryptomeria japonica* (Japanese cedar) and *Chamaecyparis obtusa* (Japanese cypress)—is a major public health concern in Japan. The number of patients seeking medical care during the pollen season, particularly those with cedar pollinosis, is associated with airborne pollen concentrations (1). Therefore, accurate monitoring of not only the total amount of airborne pollen but also the taxonomic composition of pollen in the atmosphere is of substantial clinical and social importance (2–7).

In Japan, spring pollen is monitored at numerous sites using both conventional and automated methods. Many public health centers and research institutions use the Durham gravimetric method, in which airborne particles settle passively onto Vaseline-coated slides. This method allows taxon-level identification by microscopic observation and remains useful during the spring season, when multiple pollen species coexist. However, it requires 24-h slide exposure, staining, and manual counting by trained personnel, making it labor-intensive and unsuitable for real-time assessment. In parallel, private meteorological companies have provided near real-time pollen information using laser light-scattering devices, including systems such as the KH3000, which was widely used in Japan for operational pollen monitoring. These automated devices offer clear advantages in timeliness, but they may miscount non-pollen particles, such as dust, when particle size and optical properties overlap with those of pollen. Under favorable conditions with high pollen levels and low atmospheric dust, the correlation with the Durham method can exceed 0.7 (8). More recently, near real-time automated pollen monitoring has further expanded in Japan through private meteorological companies, although reliable discrimination between pollen and non-pollen particles and taxon-level identification remain difficult in routine field monitoring. Laser-induced fluorescence has improved the possibility of taxon-level discrimination, although practical separation of cedar and cypress pollen in routine field monitoring remains challenging (9–11).

These limitations are particularly relevant when monitoring reliability is most vulnerable, such as in the early and late stages of the pollen season. During these periods, airborne pollen counts are relatively low, and misclassification of dust or other particles can have a greater influence on observed trends. In regions where multiple pollen taxa and environmental particles coexist, improving particle-level classification may help support the interpretation and reliability of both conventional slide-based monitoring and automated real-time surveillance (12).

Recent advances in pollen image analysis have demonstrated that combining morphological and textural features with support vector machines (SVMs) can provide reproducible and robust classification performance (43). However, many previous studies on machine-learning-based pollen classification have relied on idealized or laboratory-prepared pollen grains (13, 14), which limits their direct applicability to routine environmental monitoring. In real-world samples, microscopic fields often contain debris, air bubbles, non-target pollen taxa, and burst or deformed pollen grains produced by humidity or rainfall (15). Such burst-related particles including exine fragments, and released cytoplasmic material are also clinically relevant in allergic disease (16, 17). These features make classification substantially more difficult, but also more relevant for practical aerobiological use.

In Nagano Prefecture, Japan, a collaborative spring pollen-monitoring network led by Shinshu University has been established in cooperation with community otolaryngologists and local schools. This network uses the Durham method with daily slide replacement. Because the highland geography of Nagano Prefecture produces region-specific pollen profiles, species-level identification is important for interpreting local pollen exposure. Although the collection of slides from multiple sites provides broad regional coverage, reliance on manual counting increases personnel burden and may introduce inter-observer variability.

Against this background, the present study aimed to establish a reproducible and interpretable classification workflow for real-world Durham-slide images obtained in community-based monitoring. Specifically, we extracted individual particle images from environmental Durham slides and evaluated whether machine-learning methods based on handcrafted morphological features could distinguish cedar pollen, cypress pollen, burst forms, and dust-like particles. By focusing on routinely acquired microscopic images rather than idealized specimens, we sought to provide a practical methodological basis for improving the reliability of pollen monitoring in real-world settings.

## 2 Materials and methods

### 2.1 Study design and slide sources

The aim of this study was to establish a methodological proof-of-concept for automated classification of real-world Durham-slide images. Routine Durham slides collected by collaborating research institutions were sent to our clinic laboratory for standardized staining and image analysis. Slides were obtained from Matsumoto City (March 27, March 28 and April 15, 2024) and from Azumi Elementary and Junior High School (March 21 and March 24, 2025). Slides were used to increase the number of analyzable particle images under a standardized staining protocol; comparison across sites or sampling dates was not an objective of this study.

### 2.2 Durham slides and staining procedure

Pollen samples were collected using the Durham gravimetric method. Vaseline-coated glass slides were placed outdoors for 24 h to capture the naturally settling airborne pollen. Pollen on the collected slides was stained with gentian violet gelatin. The staining medium was prepared by gently heating and dissolving gelatin (10 g), glycerin (60 mL), distilled water (35 mL), 0.1% gentian violet alcohol solution (1 mL), and liquid phenol (0.5 mL). The solution was then transferred to a petri dish and cooled for storage. A small amount of staining jelly was placed on a slide containing pollen, melted by gentle heating, and mounted on a cover glass. After confirming that the pollen grains were adequately stained, a microscopic examination was performed. All slides were processed using the same staining protocol at our clinic laboratory to reduce procedural variability.

### 2.3 Microscopic observation and image acquisition

Slides were examined using a Zeiss Axiolab 5 optical microscope (Carl Zeiss, Oberkochen, Germany) equipped with an EC Plan-NEOFLUAR 40×/0.75 objective lens and PI 10×/22 eyepieces. Images were captured using a Zeiss Axiocam 208 color camera (Carl Zeiss, Oberkochen, Germany) at a 400× total magnification. Each image was recorded at 4 K resolution (3,840 × 2,160 pixels).

### 2.4 Image processing and particle cropping for dataset construction

Image processing was performed using Python 3.11.12 and OpenCV 4.10.0. Circular pollen regions were detected using the Hough Circle Transform technique (44). The detected center coordinates were used to crop a 512 × 512-pixel region around each pollen grain. A higher image resolution was selected based on prior literature indicating that resolution strongly influences model performance (18, 19). To avoid duplicate detections, clustering was performed using the DBSCAN algorithm (ver. 1.6.1). The final manually labeled dataset contained five classes: cedar pollen (internal label: cedar), 592 images; cypress pollen (internal label: cypre), 292 images; burst cedar pollen (internal label: cedar_brst), 253 images; burst cypress pollen (internal label: cypre_brst), 92 images; and dust and miscellaneous artifacts, 251 images, yielding a total of 1,480 cropped images. Here, the terms in parentheses denote the internal class labels used consistently in the analysis pipeline, figures, and repository. No formal *a priori* minimum number of images per class was imposed, because this study was designed as a proof-of-concept using real-world routine Durham slides rather than a pre-balanced benchmark dataset. Instead, all analyzable cropped particles obtained under the standardized protocol were reviewed and assigned to classes, and class imbalance was addressed at the evaluation stage by emphasizing macro-F1, balanced accuracy, and class-wise performance.

Burst pollen was handled as an explicit class rather than merged with intact pollen because ruptured grains are routinely encountered in practical slide reading and may show morphology distinct from intact grains. Including burst pollen as separate classes was intended to make the classification task more realistic and to prevent artificial simplification of the dataset. In addition, a dust/miscellaneous artifact class was included to reflect non-pollen particles and staining-related noise encountered in routine environmental slides.

Class labels were assigned by expert visual inspection by otolaryngologists experienced in pollen morphology. Representative examples of the five classes and their major morphological characteristics are shown in Supplementary Figure S1 to aid interpretation by non-specialist readers. Because multiple cropped images were obtained from the same slide, the images are not strictly independent; therefore, the present evaluation should be interpreted as a methodological proof-of-concept using real-world Durham-slide images. Per-class sample counts are summarized in Table 1. An overview of the analytical workflow is provided in Supplementary Figure S2.

**Table 1:** Per-class sample counts.

Classification	Cedar	Cypress	Burst cedar	Burst cypress	Dust/artifacts
Internal label	“cedar”	“cypre”	“cedar_brst”	“cypre_brst”	“dust”
*N*	592	292	253	92	251

The table summarizes the number of cropped particle images assigned to each analytical class: intact cedar pollen, intact cypress pollen, burst cedar pollen, burst cypress pollen, and environmental debris (dust). Terms in parentheses indicate the internal class labels used consistently in the analysis pipeline, figures, and repository.

### 2.5 Feature engineering and definition of the core-9 feature set

Eleven morphological and textural image features were initially designed as candidate predictors for machine-learning classification.

1. Radius (radius): Direct radius obtained from the Hough Circle Transform.
2. Circularity (circularity): 4*π*·Area/Perimeter^2^ is commonly used in particle and cellular morphology analysis.
3. Inscribed Ratio (inscribed_ratio): Ratio of the largest inscribed circle radius to the Hough-detected ideal circle radius, which evaluates compactness and internal filling.
4. Area (pixels) (area_px): Area of the segmented pollen region after grayscale conversion and binary thresholding.
5. Centroid Shift (centroid_shift): Distance between the Hough-based center and true centroid of the binarized pollen mask—analogous to PET-based heterogeneity indices.
6. Fractal Dimensions (fractal_dim): Estimated using the Box-Counting method to quantify contour complexity.
7. Spike Count (spike_cnt): Edge detection via Canny was followed by a probabilistic Hough transform to quantify the radial linear structures.
8. Radial Gradient (radial_grad): Intensity difference between central and peripheral regions.
9. Edge Density (edge_density): Mean intensity of Canny edge pixels normalized by region size.
10. Texture Entropy (texture_entropy): Entropy of local binary patterns, representing internal texture variation.
11. Convex Defect Ratio (convex_def_ratio): (A_hull − A_obj)/A_hull (=1 − A_obj/A_hull). A_obj: mask area; A_hull: convex hull area. Range 0–1; higher values indicate more concave/irregular boundaries (sensitive to indentations/deformation).

These features were selected to capture class-relevant differences in particle size, shape regularity, surface texture, contour complexity, and internal intensity distribution. In practical terms, intact cedar and cypress pollen, burst pollen, and non-pollen artifacts often differ along these morphological dimensions, which motivated the feature design.

Standardization was implemented within each training fold using StandardScaler in the model pipeline to prevent leakage. To construct a robust and nonredundant feature set, four criteria were evaluated: (i) redundancy, assessed using Pearson's correlation (|*r*| > 0.85) and variance inflation factor VIF (thresholds of >5 and, in sensitivity checks, >10); (ii) predictive contribution, assessed using permutation importance and drop-column analysis; (iii) stability, assessed by consistency of feature importance across 5-fold cross-validation; and (iv) biological validity, assessed by expert review based on morphological plausibility.

For drop-column analysis, we compared the macro-F1 score obtained with nested 5-fold cross-validation after removing one candidate feature at a time from an 11-feature set, using identical splits and hyperparameter search settings across feature sets. Based on these criteria, fractal_dim was excluded due to low biological plausibility and high VIF, and edge_density was preferentially excluded over spike_cnt because drop-column results indicated that spike_cnt carried greater unique predictive information.

Thus, a refined core set of nine features (core-9) was defined as follows: radius, centroid_shift, circularity, spike_cnt, texture_entropy, area_px, radial_grad, inscribed_ratio, and convex_def_ratio.

### 2.6 Model training and validation

Classification was performed using a support vector machine (SVM) with a radial basis function (RBF) kernel implemented using the scikit-learn pipeline: StandardScaler → SVC (kernel=“rbf”, probability = True). In this study, training refers to fitting the model parameters using images in the training folds, whereas validation refers to evaluating candidate hyperparameter settings on held-out subsets within the training data during model selection. These procedures were separated to reduce overly optimistic performance estimation.

To ensure rigorous evaluation and minimize optimistic bias, nested stratified cross-validation was used as follows: Outer loop: StratifiedKFold (*k* = 5, shuffle = True, random_state = 42) and Inner loop: StratifiedKFold (*k* = 5, shuffle = True, random_state = 1). All preprocessing steps, including feature scaling, were performed within the training folds of the nested cross-validation pipeline.

The hyperparameter grid was as follows: C ∈ {0.3, 1, 3, 10, 30}, gamma ∈ {0.003, 0.01, 0.03, “scale”}, and class_weight ∈ {None, “balanced”}. The primary evaluation metric was macro-F1 score (mean ± SD across outer folds). Secondary metrics included balanced accuracy, class-wise F1, receiver operating characteristic (ROC) curves, area under the ROC curve (AUC), precision-recall (PR) curves, and area under the PR curve (AUPRC).

For readers less familiar with machine-learning terminology, precision indicates the proportion of images predicted to belong to a given class that were actually correct, whereas recall indicates the proportion of truly belonging images that were successfully detected by the model. Because some classes were relatively infrequent, PR curves were additionally emphasized as recommended for imbalanced classification problems.

Out-of-fold (OOF) predictions from all outer folds were aggregated to construct a confusion matrix, one-vs.-rest ROC curves, PR curves, and permutation importance plots. Model performance was primarily summarized using macro-F1, with ROC-AUC and PR-AUC reported as complementary measures.

### 2.7 Reproducibility

All analyses were implemented using Python v. 3.11.12, scikit-learn (pipeline, GridSearchCV), and OpenCV v. 4.10.0. The full reproducible pipeline (image detection/cropping, feature extraction, and SVM evaluation) is archived on Zenodo (version v1.0.2): doi: 10.5281/zenodo.18130679. Class labels (type) were assigned by expert visual inspection and are intentionally not automated in the repository.

## 3 Results

### 3.1 Feature selection and definition of the core-9 feature set

**Figure 1:**
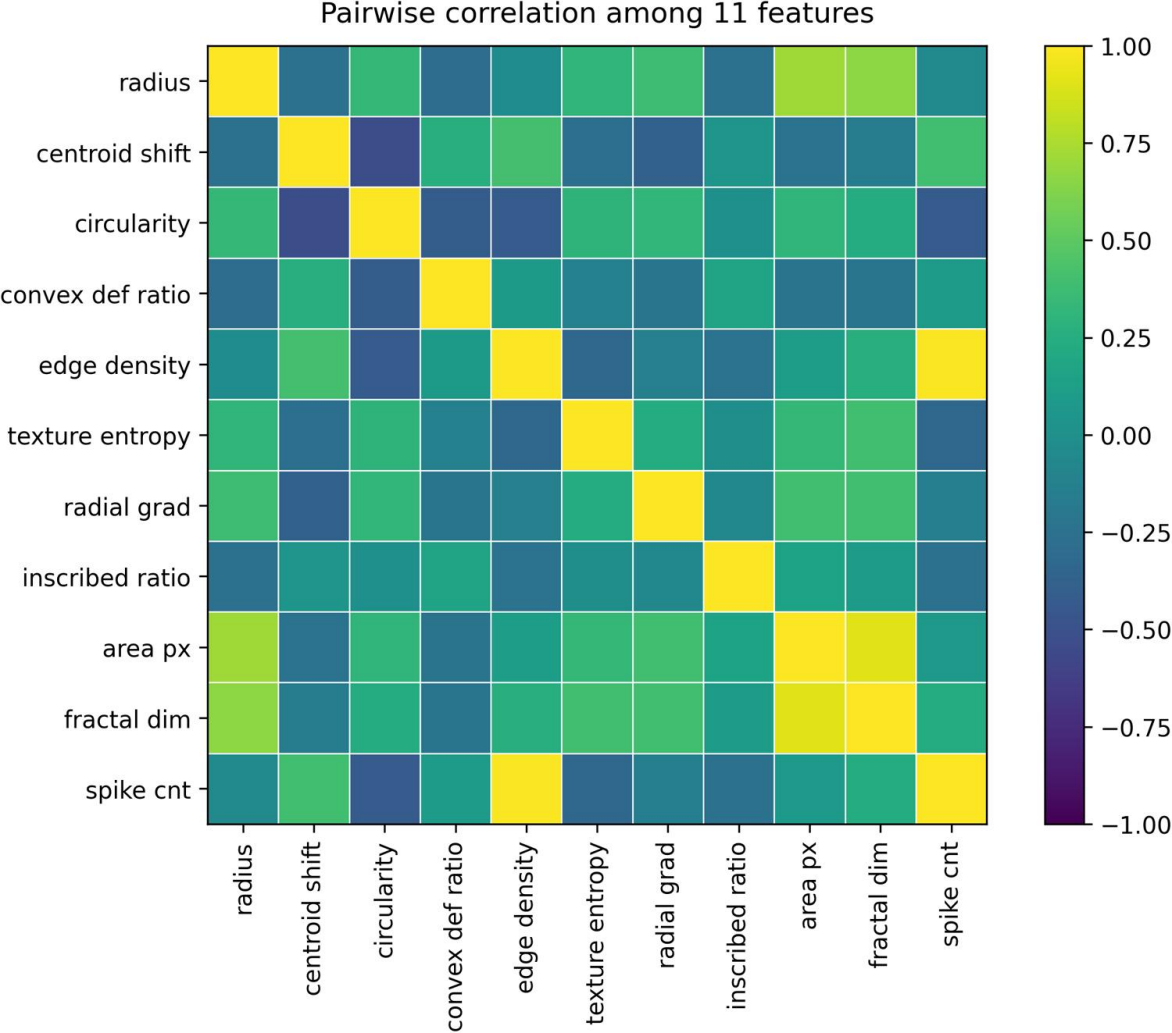
Pairwise Pearson's correlation matrix for the 11 candidate morphological and textural features. Each cell represents the correlation coefficients between two features, with color intensity indicating the direction and magnitude of the correlation (yellow: strongly positive; blue: strongly negative). The diagonal values are 1.0 by definition. This analysis was used to assess multicollinearity prior to model construction.

**Figure 2:**
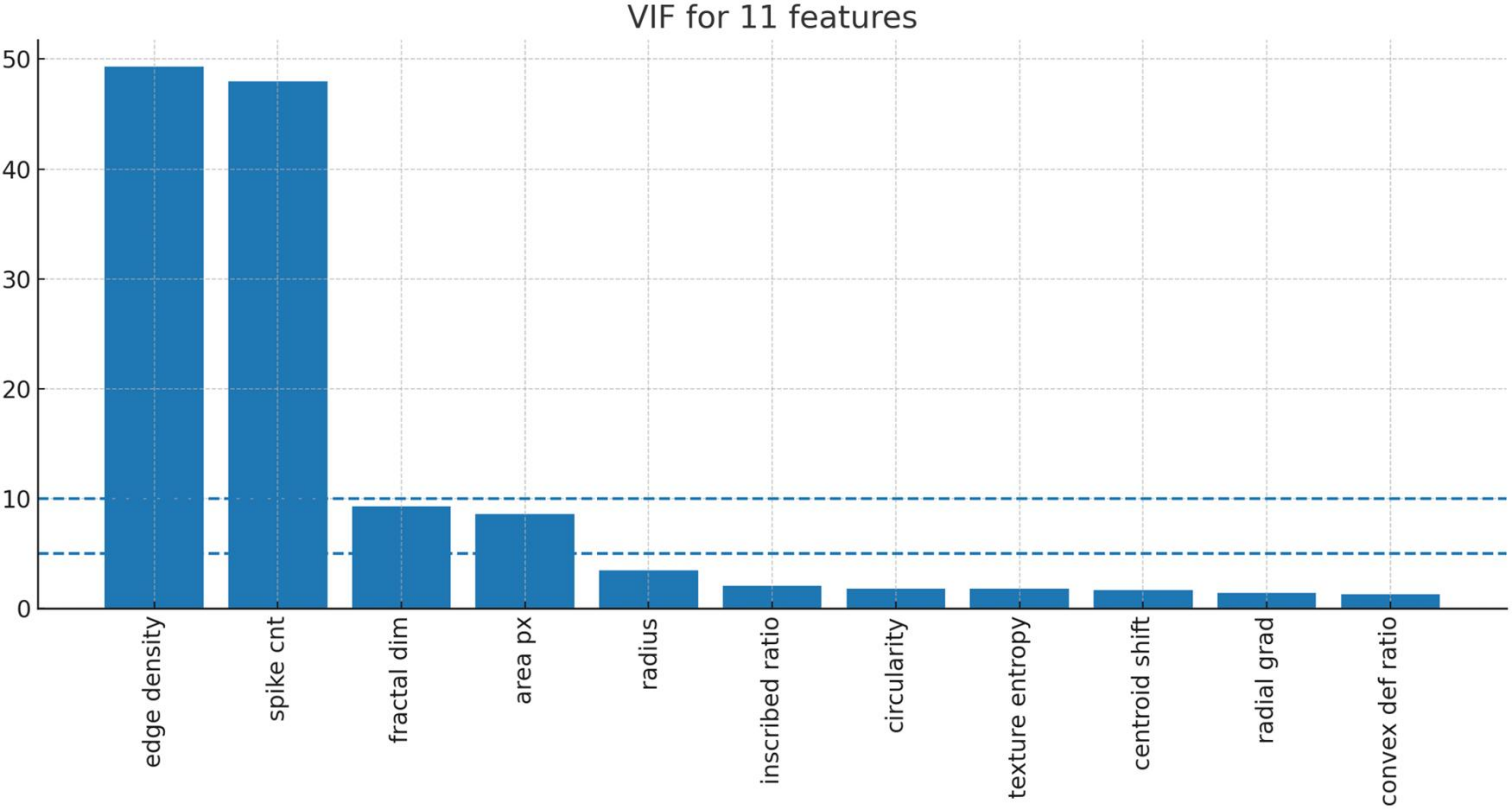
Variance inflation factor (VIF) values for the 11 candidate features. The bars represent the VIF values calculated by regressing each feature against all remaining features (after Z-score normalization). The dashed lines indicate common thresholds (VIF = 5 and VIF = 10).

To define the final feature set, we first examined relationships among the 11 candidate features. Pairwise correlation analysis revealed strong correlations between edge_density and spike_cnt, and between fractal_dim and area_px (Figure 1). Consistent with this, variance inflation factor (VIF) analysis showed marked multicollinearity for edge_density (49.3) and spike_cnt (47.9), while fractal_dim (9.31) and area_px (8.57) also showed elevated values (Figure 2).

We then performed a column-ablation analysis to evaluate the contribution of individual features to classification performance. Four feature sets were tested: A) all 11 features, B) removal of fractal_dim, C) removal of spike_cnt, and D) removal of edge_density. The corresponding macro-F1 scores (mean ± SD across outer folds) were 0.816 ± 0.019, 0.828 ± 0.031, 0.818 ± 0.007, and 0.823 ± 0.030, respectively (Table 2). Removal of fractal_dim had little effect on model performance, whereas removal of spike_cnt caused a larger decrease in macro-F1 than removal of edge_density. Based on multicollinearity, performance retention, and biological interpretability, fractal_dim and edge_density were excluded, and the remaining nine variables were defined as the core-9 feature set. Performance was preserved after exclusion of these two variables.

**Table 2:** Performance comparison of feature sets evaluated using nested 5-fold cross-validation.

Group name	Set	*k*	f1 macro mean	f1 macro sd
A	A: 11_features	11	0.816444	0.019
B	B: no_fractal	10	0.828198	0.031185
C	C: no_spike	10	0.818133	0.007177
D	D: no_edge	10	0.822708	0.029988
E	E: core9	9	0.833257	0.024936

The feature sets included A) all 11 features; B) excluding *fractal_dimensions*; C) excluding *spike_cnt*; D) excluding *edge_density*; and E) Core-9 (excluding *fractal_dimensions* and *edge_density*). Macro-F1 scores are shown as mean ± standard deviation. Core-9 reduced multicollinearity, while maintaining a performance comparable to that of the full 11-feature set. The macro-F1 scores for A and E were further compared using a paired t-test across the outer folds (two-sided, *df* = 4).

### 3.2 Overall classification performance of the final model

Using the core-9 feature set, model performance was evaluated with nested 5-fold stratified cross-validation. The macro-F1 score, calculated as the mean ± SD across the outer folds, was 0.833 ± 0.025 (95% CI: 0.802–0.864; df = 4). The overall accuracy calculated from aggregated out-of-fold (OOF) predictions was 0.863.

**Figure 3:**
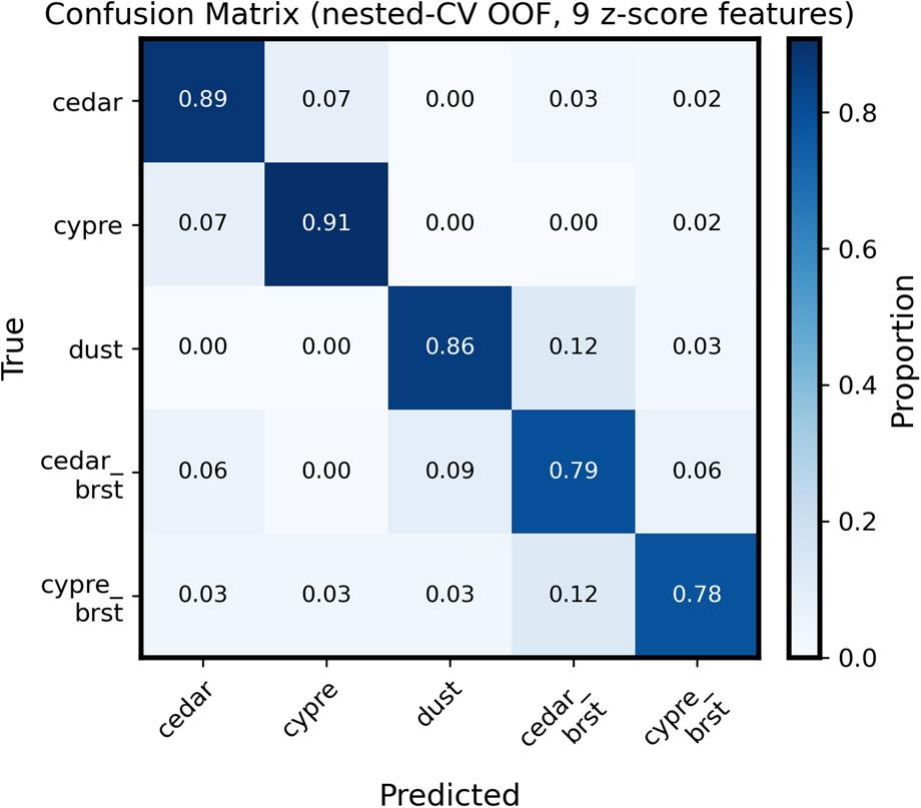
Confusion matrix of the nested 5-fold cross-validation (out-of-fold predictions) using the core-9 feature set. Each cell represents the proportion of samples assigned to each predicted class, normalized by the number of samples in the true class (row-normalized; equivalent to class-wise recall). Diagonal values indicate correct classification rates for each class, whereas off-diagonal values indicate misclassifications. Misclassifications were most frequently observed between cedar and cypress, and between dust or cypre_brst and cedar_brst, reflecting morphological similarity among these categories.

The confusion matrix constructed from aggregated OOF predictions is shown in Figure 3. High diagonal values were observed across all five classes, indicating good overall discrimination. Misclassifications were observed mainly between cedar and cypress, and from dust or burst cypress to burst cedar. These errors were concentrated in class pairs with partially overlapping morphological characteristics.

### 3.3 Class-wise discriminative performance

**Figure 4:**
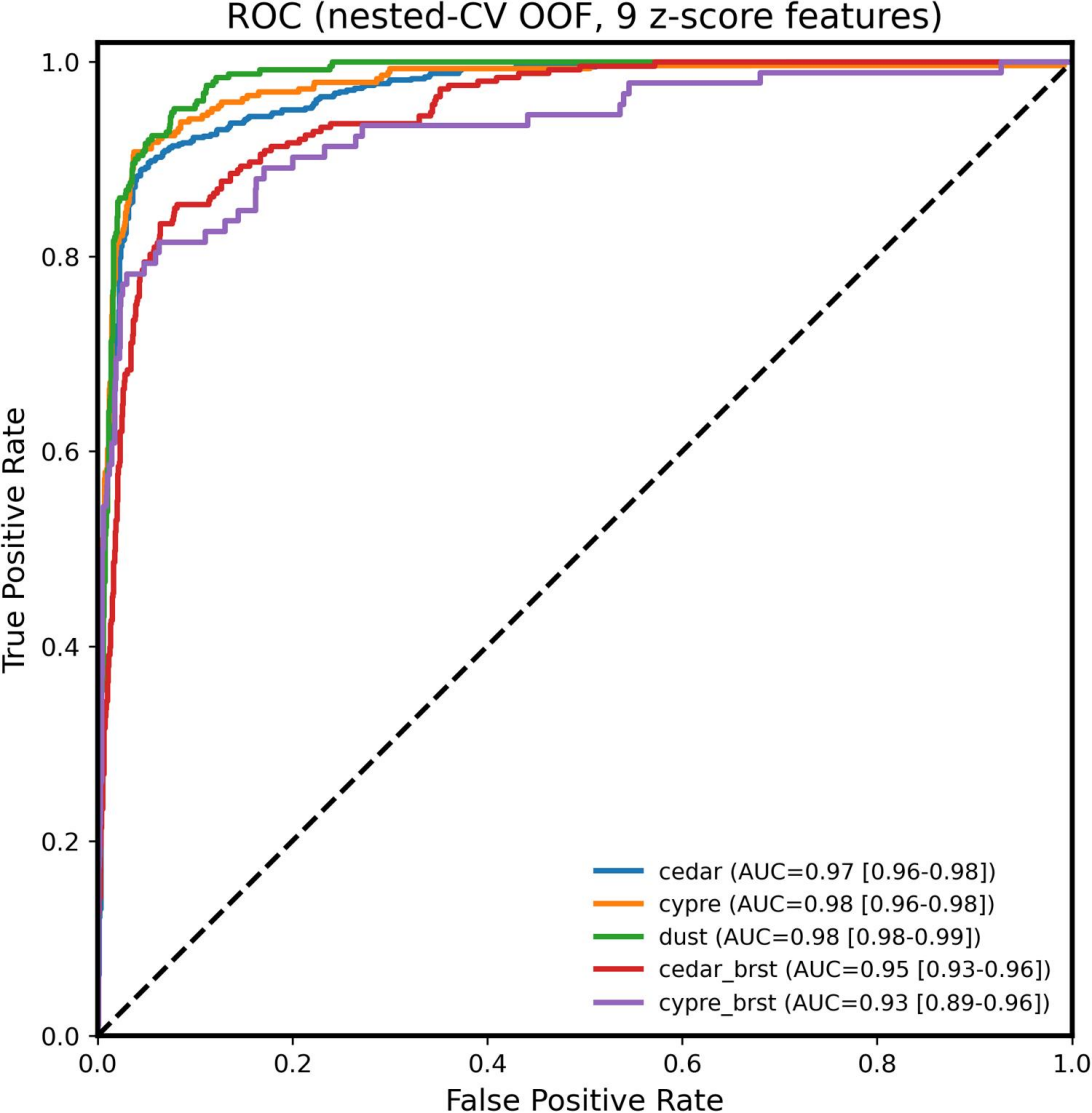
ROC (receiver operating characteristic) curves for the five pollen classes using the core-9 feature set. Curves represent one-vs.-rest ROC analysis for each class, with AUC values shown in the legend. All classes demonstrated high discriminative performance (AUC: 0.92–0.98), with intact pollen classes (cedar, cypress, dust) showing the highest values.

Receiver operating characteristic (ROC) analysis demonstrated high class-wise discriminative ability (Figure 4). The one-vs.-rest area under the curve (AUC) values were 0.97 (95% CI: 0.96–0.98) for cedar, 0.98 (95% CI: 0.96–0.98) for cypress, 0.98 (95% CI: 0.98–0.99) for dust, 0.95 (95% CI: 0.93–0.96) for burst cedar, and 0.93 (95% CI: 0.89–0.96) for burst cypress.

**Figure 5:**
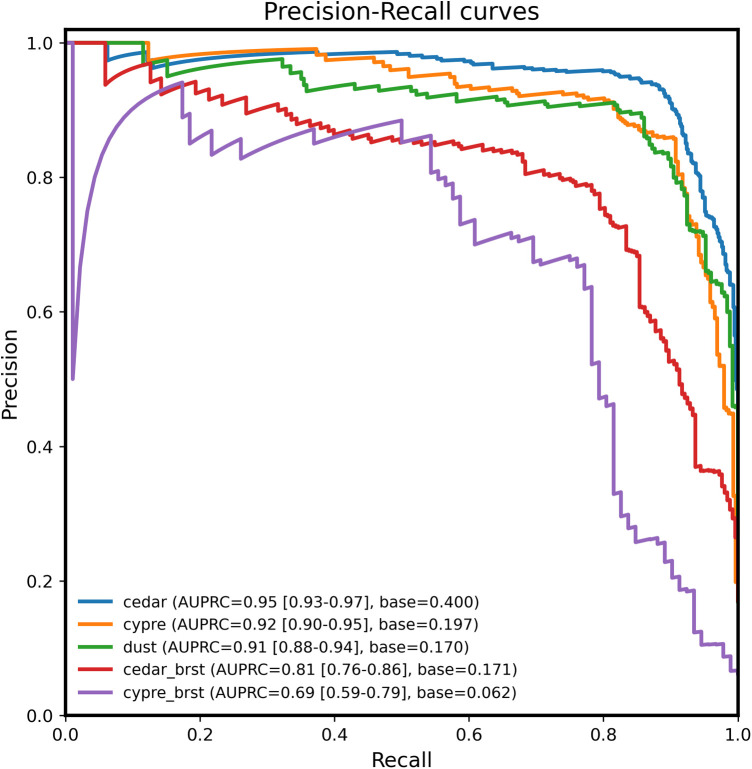
Precision–recall (PR) curves for the five pollen classes using out-of-fold predictions. Curves display one-vs.-rest PR analysis with AUPRC values shown in the legend. Micro- and macro-averaged AUPRCs were 0.90 and 0.85, respectively. The intact pollen classes showed high precision across recall levels, whereas the burst classes (cedar_brst and cypre_brst) exhibited lower values owing to sample imbalance and structural heterogeneity.

Precision-recall (PR) analysis showed similarly strong performance for the more abundant intact classes (cedar, cypress, and dust) (Figure 5). In contrast, the burst classes showed lower precision and recall. The area under the precision-recall curve (AUPRC) was 0.81 (95% CI: 0.76–0.85) for burst cedar and 0.69 (95% CI: 0.59–0.79) for burst cypress. Both values exceeded their respective class-prevalence baselines (0.17 and 0.06). The lower PR performance of the burst classes was consistent with their greater morphological heterogeneity and smaller sample sizes.

### 3.4 Feature importance in the final model

**Figure 6:**
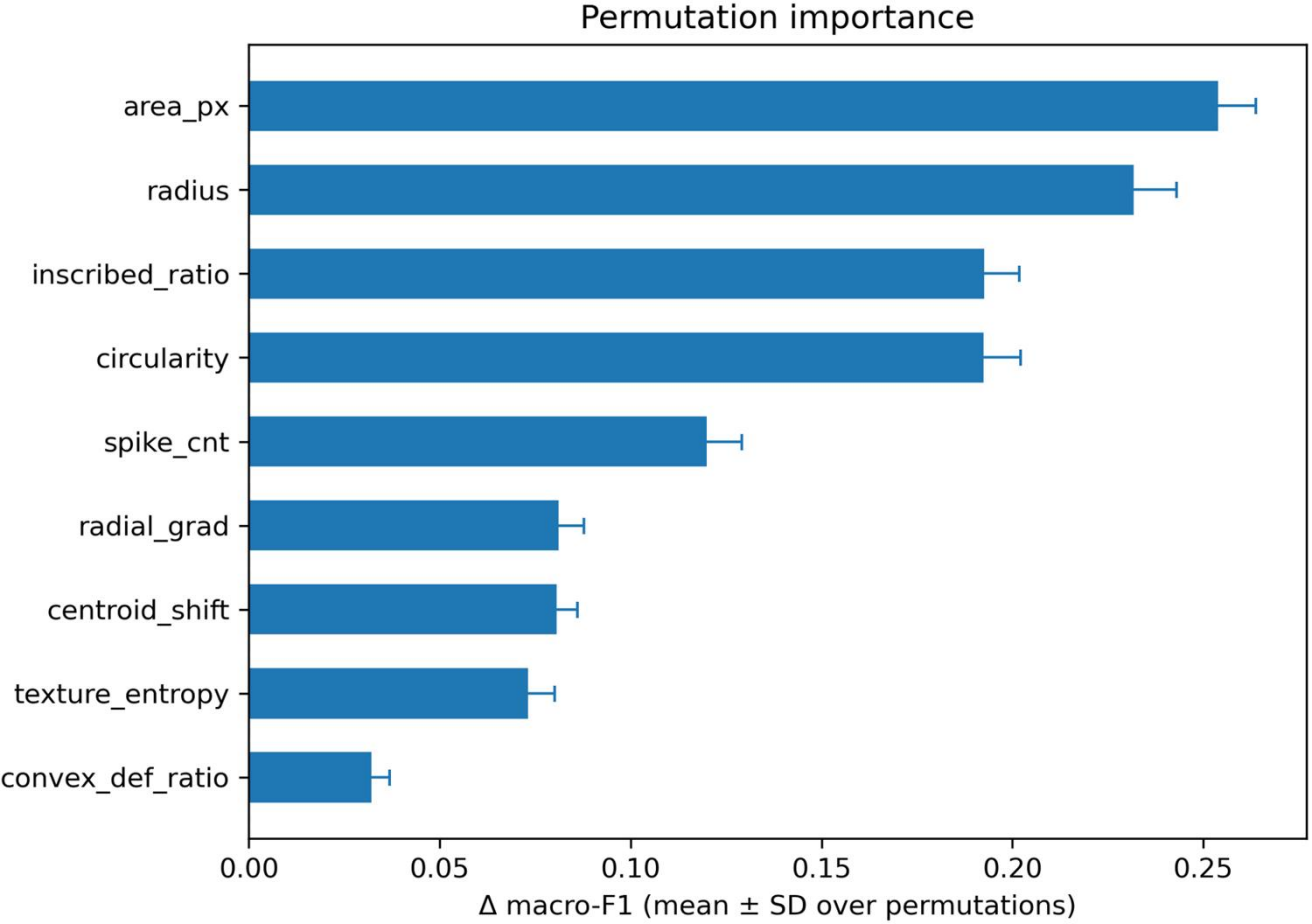
Permutation importance of the nine selected (core-9) features in the nested cross-validation model. Bars represent the mean decrease in the macro-F1 score when each feature was permuted, with error bars indicating the standard deviation. Size-related features (*area_px* and *radius*) showed the highest importance, followed by shape features (*inscribed_ratio* and *circularity*). Texture-related features demonstrated lower contributions.

The permutation importance analysis of the final core-9 model is shown in Figure 6. Among the nine variables, area_px and radius produced the largest decreases in performance when permuted, followed by the shape-related features inscribed_ratio and circularity. In contrast, texture-related variables such as texture_entropy and convex_def_ratio showed smaller contributions.

## 4 Discussion

This study demonstrated that machine-learning-based classification using interpretable morphological features can distinguish cedar, cypress, burst pollen, and environmental debris from real-world microscopic slides prepared using the Durham method. In contrast to previous studies that relied mainly on idealized laboratory-prepared pollen samples (13, 14, 20–22), our dataset consisted of heterogeneous field-derived images that included debris, air bubbles, other pollen taxa, and structurally deformed burst pollen. This heterogeneity more closely reflects routine environmental aerobiology. Within this setting, the core-9 feature set achieved stable performance under nested cross-validation (macro-F1 = 0.833; 95% CI: 0.802–0.864), supporting the feasibility of this workflow as a methodological proof-of-concept for real-world Durham-slide images.

**Figure 7:**
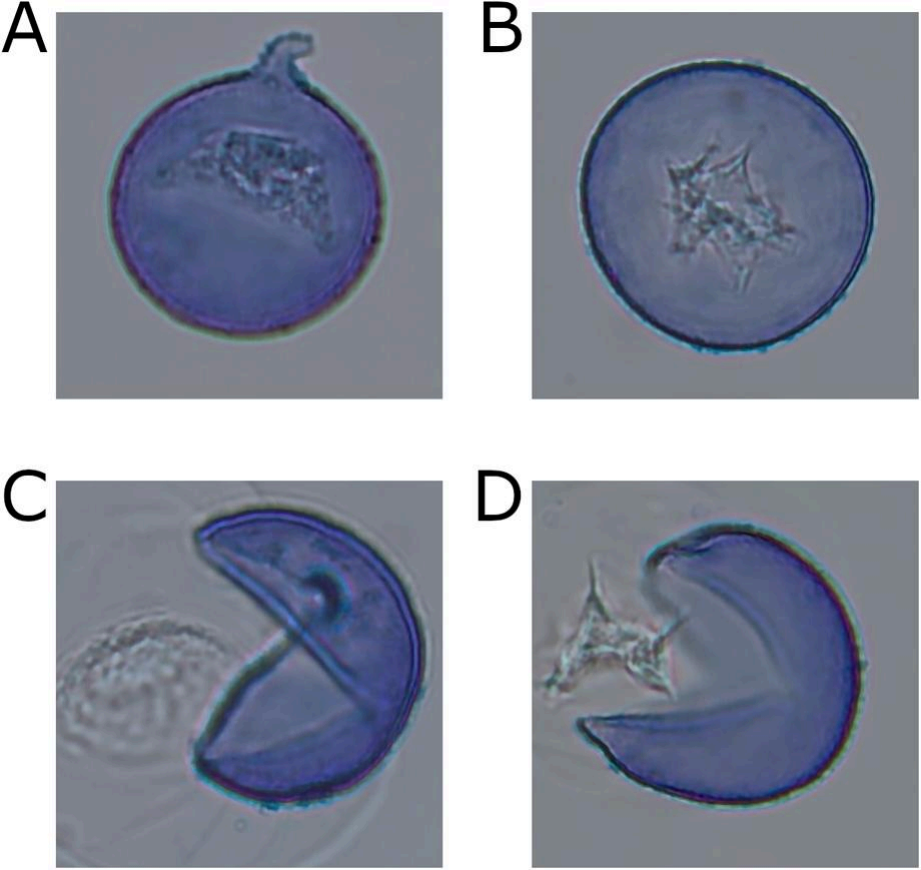
Representative images of intact and burst pollen of Japanese cedar and Japanese cypress. **(A)** Intact *Cryptomeria japonica* (Cedar) pollen. The protruding structure is the germinal furrow, with characteristic internal cytoplasmic patterns concentrated near the aperture. **(B)** Intact *Chamaecyparis obtusa* (Cypress) pollen showing a smooth spherical shape and a centrally located internal pattern distinct from Cedar. **(C)** Burst Cedar pollen with ruptured exine and exposed cytoplasmic and nuclear contents. **(D)** Burst Cypress pollen with ruptured exine and distinct cytoplasmic material morphology compared to Cedar.

A key finding of the present study was the strong contribution of size-related features, particularly area_px and radius, which showed the highest permutation importance in the final model. This finding is consistent with established differences in pollen size between *Cryptomeria japonica* and *Chamaecyparis obtusa* (23, 24). Burst pollen typically exhibits swelling or asymmetric disruption due to water uptake (Figure 7) (15), resulting in enlarged radii and irregular projected areas that may help distinguish intact pollen from burst pollen and dust. This also supports the decision to treat burst particles as a separate analytical category rather than merging them with intact pollen. A consistent classification scheme for intact pollen, burst pollen, and artifacts is important for reproducibility across sites and future model updating. Shape-related variables, including circularity, inscribed_ratio, convex_def_ratio, and centroid_shift, also contributed meaningfully to classification. These descriptors captured deviations from the approximately spherical geometry of intact cedar and cypress pollen. When pollen bursts, the exine collapses asymmetrically, and the cytoplasmic contents extrude irregularly, leading to reduced circularity and compactness together with increased contour irregularity. The biological plausibility of these features explains their consistent performance across folds (25). By contrast, texture-related descriptors contributed less than expected. Although internal cytoplasmic patterns may differ between pollen types, their visibility depends strongly on pollen orientation and staining conditions. These findings indicate that some errors are concentrated in morphologically ambiguous classes, particularly under low-count conditions. Similar orientation-dependent limitations of texture-based descriptors have been reported in radiological and cytological imaging (26–29), which is consistent with the relatively modest contribution observed here.

The comparison between spike_cnt and edge_density provided additional insight into feature selection. Although these variables were highly correlated, the ablation analysis showed that removal of spike_cnt produced a larger reduction in macro-F1 than removal of edge_density, indicating that spike_cnt retained more unique predictive information. Cedar pollen possesses germinal furrows that can appear as radial linear structures depending on orientation (30), and probabilistic Hough line detection may preferentially capture such structures. In contrast, edge_density is more sensitive to noise introduced during staining, thresholding, and segmentation (31). These considerations support the exclusion of edge_density while retaining spike_cnt in the final feature set.

The exclusion of fractal dimensions was also supported by both biological and methodological considerations. Fractal analysis has shown utility in other biomedical imaging contexts, such as tumor morphology (32, 33), but pollen exine architecture does not exhibit clear self-similarity over multiple spatial scales. In addition, fractal dimension is sensitive to imaging resolution and segmentation thresholds (34), both of which can vary in field microscopy. Taken together with the minimal performance change seen in the ablation analysis, these findings support the decision to exclude fractal_dim from the final core-9 feature set.

Classification of burst pollen remained the most challenging task. Burst pollen is clinically relevant because it can release allergen-carrying fragments, including exine fragments and dispersed cytoplasmic material (16, 17). Its morphology varies widely, with fragmented exines, irregular swelling, and heterogeneous dispersal of internal contents (35). This variability likely reduces the stability of shape-based descriptors and makes separation from other irregular particles more difficult. In the present study, class-wise ROC-AUC values remained high, whereas PR curves showed lower precision and recall for the burst classes. This discrepancy is consistent with the combined effects of class imbalance and morphological heterogeneity. Accordingly, future work should evaluate calibrated probabilities to optimize thresholding and reduce false negatives in low-count settings. Nevertheless, the AUPRC values for burst cedar and burst cypress remained above their class-prevalence baselines, suggesting that the selected geometric features still captured meaningful structure even under these challenging conditions.

Compared with recent high-performance approaches such as imaging flow cytometry (30, 36), microfluidic impedance/optical systems (37), and multimodal deep-learning approaches (9), the present method emphasizes interpretability and compatibility with routine microscopy-based monitoring. Deep learning may outperform classical machine-learning approaches on highly curated datasets, but interpretability is limited and large annotated datasets are generally required (38). In contrast, interpretable geometric descriptors provide a more transparent account of classification behavior, which may be particularly valuable in environmental health research and community-based surveillance settings. More broadly, the present findings suggest that careful feature engineering, guided by correlation structure, VIF analysis, ablation testing, and biological interpretability, can be more advantageous than simply increasing feature complexity.

Limitations

This study has several limitations. First, the number of source slides was limited (*n* = 5), and the slides were selected to increase the number of analyzable particle images rather than to enable comparisons across sites, dates, or environmental conditions. Second, multiple cropped images were derived from the same slide and were therefore not fully independent at the slide level. Because only five source slides were available, slide-level variability could not be robustly quantified in the present study. As a result, the present findings should be interpreted primarily as a methodological proof-of-concept demonstrating feasibility on real-world Durham-slide images, rather than as definitive evidence of generalizability across broader monitoring contexts. A slide-level cross-validation design would be valuable in future studies, but it was not feasible here because the number of source slides was too small to support stable fold construction. Third, because the dataset was obtained under a limited range of imaging and preparation conditions, additional validation across seasons, locations, and staining conditions will be necessary to establish broader external validity, as these factors may alter particle appearance, staining intensity, and background noise.

Future directions

Several directions may help extend the present framework. Because burst pollen remained difficult to classify, domain-preserving augmentation and class-balancing resampling approaches may improve robustness for these morphologically heterogeneous classes (39–41). In addition, hybrid models that combined interpretable geometric descriptors with shallow convolutional neural network (CNN)-derived textural embeddings may improve performance while preserving at least partial interpretability (42). Expansion of the dataset across multiple seasons, geographic regions, and preparation conditions, together with integration into automated slide-scanning workflows, will be important for evaluating broader generalizability and strengthening real-world applicability.

## 5 Conclusion

This methodological proof-of-concept demonstrates that a curated set of interpretable morphological descriptors can support accurate machine-learning-based classification of environmental pollen, including burst pollen, using standard optical microscopy. This workflow provides a practical foundation for scalable aerobiological monitoring and for future studies linking regional pollen exposure to clinical and public health outcomes.

## Data Availability

The datasets presented in this study can be found in online repositories. The names of the repository/repositories and accession number(s) can be found below: Repository: Zenodo—DOI: 10.5281/zenodo.18446012 (Record: 18446012)Data/code link: https://zenodo.org/records/18446012GitHub (source repository): https://github.com/NS0720/pollen-svm-ifar.
