# Supplementary figures and images for: Interpretable machine learning classification of cedar and cypress pollen on routine Durham slides for environmental exposure assessment
Source: Front Allergy. 2026 May 7;7:1805985. doi: 10.3389/falgy.2026.1805985 (PMC13189800; doi:10.3389/falgy.2026.1805985)

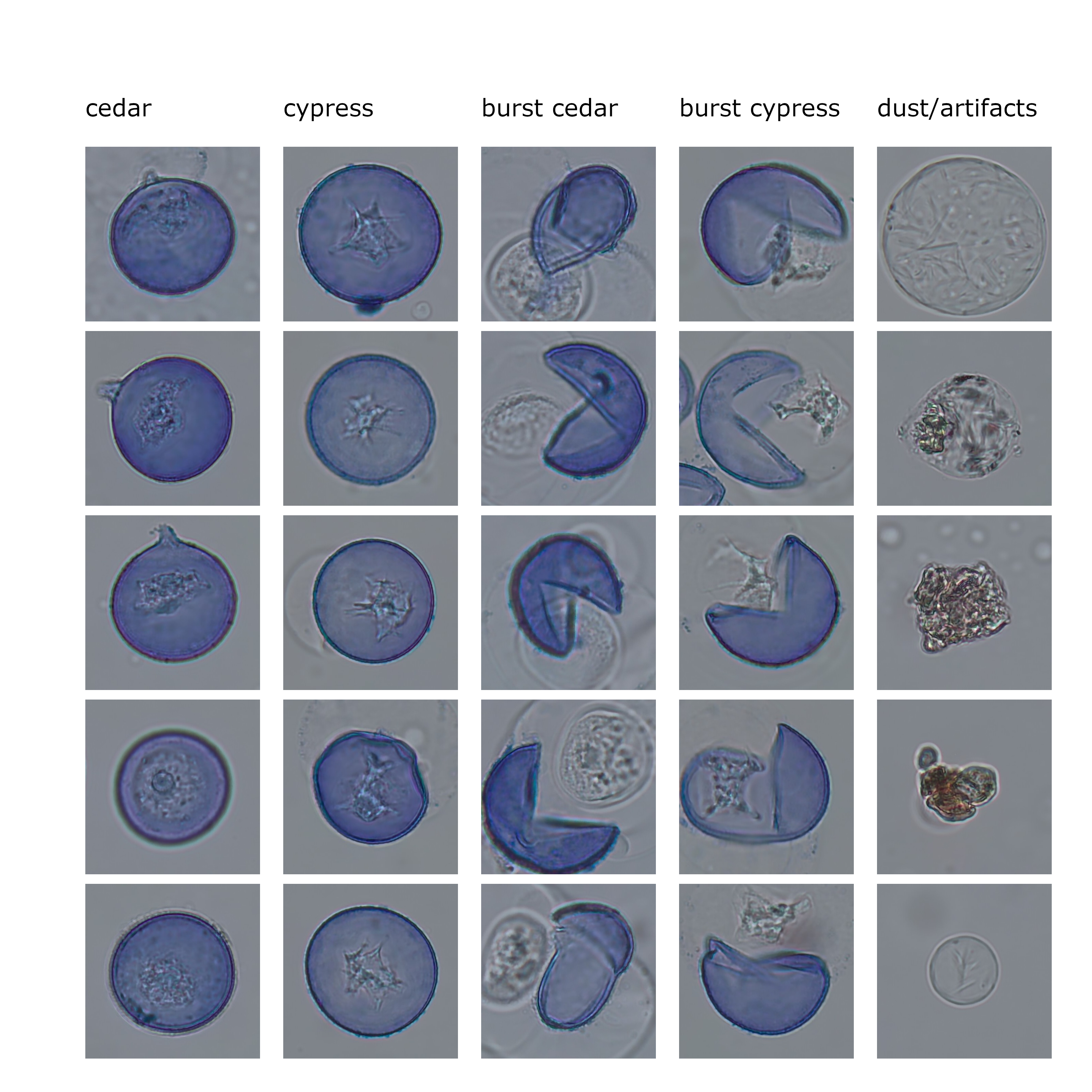

Supplement: Supplementary file 1 [file Image1.jpeg]

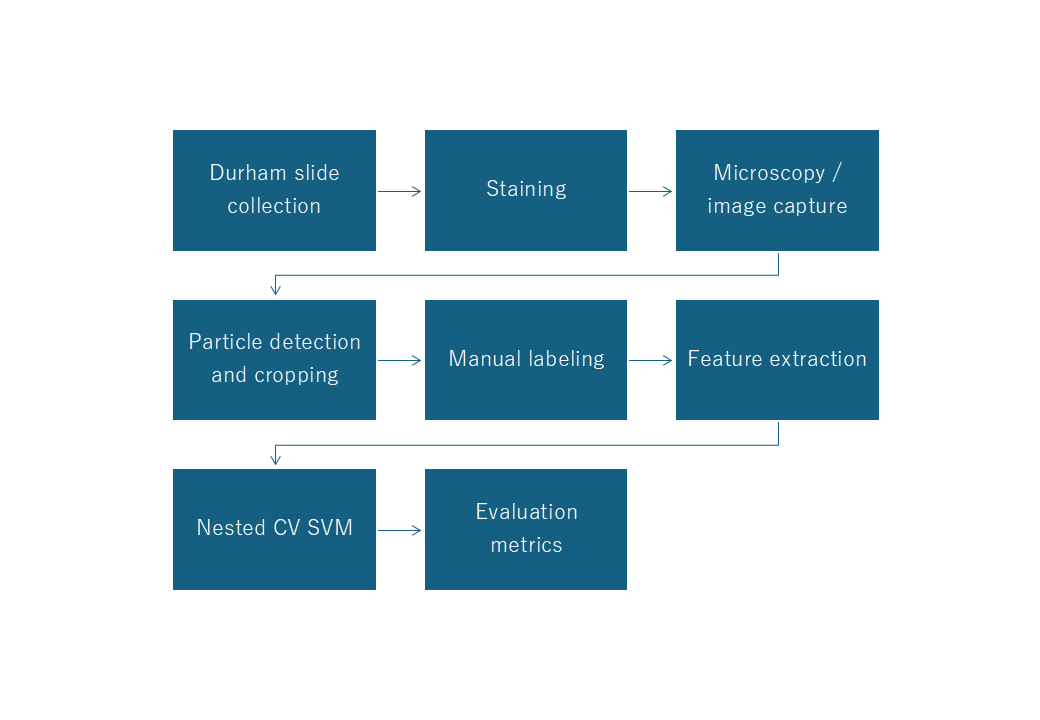

Supplement: Supplementary file 2 [file Image2.tif]
